# Anionic polymerization of *p*-(2,2′-diphenylethyl)styrene and applications to graft copolymers

**DOI:** 10.1080/15685551.2016.1231044

**Published:** 2016-09-21

**Authors:** Minglu Huang, Bingyong Han, Jianmin Lu, Wantai Yang, Zhifeng Fu

**Affiliations:** ^a^ State Key Laboratory of Chemical Resource Engineering, Beijing University of Chemical Technology, Beijing, PR China

**Keywords:** *p*-(2,2′-diphenylethyl)styrene, living anionic polymerization, macroinitiator, graft copolymer

## Abstract

Well-controlled anionic polymerization of an initiator-functionalized monomer, *p*-(2,2′-diphenylethyl)styrene (DPES), was achieved for the first time. The polymerization was performed in a mixed solvent of cyclohexane and tetrahydrofuran (THF) at 40 °C with *n*-BuLi as initiator. When the volume ratio of cyclohexane to THF was 20, the anionic polymerization of DPES showed living polymerization characteristics, and well-defined block copolymer PDPES-*b*-PS was successfully synthesized. Furthermore, radical polymerization of methyl methacrylate in the presence of PDPES effectively afforded a graft copolymer composed of a polystyrene backbone and poly(methyl methacrylate) branches. The designation of analogous monomers and polymers was of great significance to synthesize a variety of sophisticated copolymer and functionalize polymer materials.

## Introduction

Anionic polymerization is the most effective method for synthesizing homopolymers, copolymers, block polymers, and ω-functionalized polymers.[1–4] Anionic polymerization of styrene (St) is the most ideal living system and enables ready access to a variety of well-defined macromolecular architectures.[3] Introducing functional groups into St that do not interfere with the living polymerization mechanism enable the synthesis of functional polymers with precisely controlled chain structures.[4] This is essential for developing sophisticated polymers. The polar functional groups in styrene are not amenable to anionic polymerization conditions. Therefore, low temperature, −78 °C in tetrahydrofuran (THF), and appropriate protection are required.[4–7] However, alkyl- [8–10] and aryl- [10–12] substituted styrene derivatives were previously known to undergo living anionic polymerization without side reactions in hydrocarbon solvent at room temperature, which is important for the industrial production of polystyrene (PS).[3]

St with a radical initiating moiety and a polymerizable double bond, a typical radical-initiator-functionalized monomer (inimer), has recently attracted considerable attention for use in preparing hyperbranched [13–19] or highly branched [15] polymers. However, controlling the chain structure and architecture of the final polymers is difficult because the self-condensing vinyl polymerization of the inimers is unavoidable during radical polymerization for the high reactivity of the radical initiating moiety with a double bond.[13–16] The radical initiating moiety remains in a dormant state under anionic polymerization conditions, and the resulting polymers are interesting as stable intermediates of the final polymers. For example, the polymers can be used as the backbone chains for graft copolymers [17] and dendrimers [18] as well as to introduce other functions.[19]

In previous studies, monomeric and polymeric compounds with the triphenylethane (TPE) group have been reported to serve as a thermal iniferter of living radical polymerization in homogeneous systems.[20,21] To develop a new type of inimer, we introduced the polymerizable double bond into TPE to produce a new functional St, *p*-(2,2′-diphenylethyl)styrene (DPES). DPES has afore mentioned inimer-type structure, a vinyl group, and TPE as a radical initiating moiety.

To obtain PDPES with a well-defined polymer chain structure, Okuda et al. [22] synthesized it through radical and coordination Polymerizations. However, these polymerization processes were not well-controlled (PDPES yield = 7–62 wt%; polydispersity index [PDI] = 1.37–2.21), and little information has been reported concerning the anionic polymerization of DPES.

Recently, we examined the possibility of the anionic polymerization of DPES to develop a new method for synthesizing TPE containing polymers on the side chain with well-defined structures. For anionic polymerization, the solubility of the initiator and propagating species is a crucial factor. To determine the characteristics of the polymerization, the anionic polymerization of DPES was attempted under various polymerization conditions. A cyclohexane/THF mixed solvent was discovered to be particularly active for the anionic polymerization of DPES. In addition, the polymerization of methyl methacrylate (MMA) in the presence of PDPES was investigated to prepare a graft copolymer composed of a PS backbone and polymethacrylate (PMMA) branches.

## Experimental section

### Materials

All chemicals were purchased from Aldrich Chemical Company. Calcium hydride (CaH_2_), calcium chloride (CaCl_2_), methanol, and *n*-BuLi (2.5 M in cyclohexane) were used as received. *p*-Vinylbenzylchloride (p-VBC; ≥99%), diphenylmethane (≥99%), toluene (≥98%), St (≥98%), MMA (≥98%), and cyclohexane (≥98%) were refluxed over calcium hydride and distilled under dry argon. THF (≥98%) was distilled from sodium naphthalene, after it was dried over CaCl_2_ for 48 h.

DPES was synthesized through a nucleophilic substitution of the chlorine atom in *p*-VBC, which is summarized as follows. Diphenylmethane (4.8 mL, 35.0 mmol) and *n*-BuLi (14.0 mL, 35.0 mmol) were reacted at −78 °C in THF for 2 h under dry argon. *p*-VBC (5.5 mL, 35.0 mmol) was then added at −78 °C. The solution was warmed to room temperature and stirred for an additional 2 h. The crude DPES was purified and recrystallized twice in methanol (−30 °C) to obtained yield colorless needles (4.8 g, 16.9 mmol, 48%).

### Anionic polymerization of DPES

Anionic polymerization of DPES was implemented in a 50 mL glass reactor with the protection of argon, in accordance with the recipe summarized in Table 1. Firstly, well dried 50 mL glass reactors were flamed prior to use, aimed to get rid of oxygen and water. DPES was dissolved in solvent (cyclohexane, toluene, or THF) with a 10 wt% solution in the flamed reactor. Subsequently, the required amount of *n*-BuLi was then added to the reactor by using a hypodermic syringe to initiate the polymerization. The polymerization was stirred for 6 h and then terminated with methanol. The resultant polymers were precipitated into excess methanol to precipitate the polymer, which was then separated through filtration, and dried in vacuum oven to constant weight.

**Table 1.** Effect of the solvent and temperature on the anionic polymerization of DPES^a^.

No.	[DPES]_0_/[*n*-BuLi]_0_	Temperature (°C)	Solvent	Yield (wt％)	*M*_n,th_^b^ ×10^−3^	*M*_n,GPC_^c^ ×10^−3^	PDI ^d^
1	7	40	Cyclohexane	90	1.8	1.9	1.11
2	30	40	Cyclohexane	35	3.0	3.1	1.40
3	160	40	Cyclohexane	29	13.2	13.6	1.52
4	7	40	Toluene	87	1.8	2.3	1.52
5	30	40	Toluene	65	5.6	8.3	1.57
6	160	40	Toluene	60	27.3	18.1	1.54
7	7	40	THF	92	1.8	3.3	1.26
8	30	40	THF	84	7.2	27.6	1.31
9	160	40	THF	80	36.4	53.3	2.31
10	7	10	THF	82	1.8	2.4	1.31
11	7	0	THF	80	1.8	2.0	1.22
12	7	−78	THF	94	2.0	2.1	1.09

^a^ Polymerization was executed in a dry argon atmosphere at 40 °C for 6 h

^b^ *M*_n.th_: FW(C_22_H_20_) × [DPES]_0_/[*n*-BuLi]_0 _× yield(%)×10^−2^ + FW(C_4_H_9_)

^c^ *M*_n,GPC_ and

^d^ PDI were estimated using a GPC by employing PS as the standard.

### Block copolymerization of DPES and St

Block copolymerization of DPES and St was carried out in a 50 mL glass reactor with the protection of argon. The glass reactor was flamed prior to use, aimed to get rid of oxygen and water. DPES (0.3 g) was dissolved in cyclohexane (1 mL) and THF (20 mL) mixed solvent. Subsequently, *n*-BuLi (80 μL, 2.5 M) was introduced to the reactor by using a hypodermic syringe to initiate the polymerization. After stirring about 6 h at 40 °C, St (1.7 g) was then added to the reactor and the polymerization was continued for 6 h. A small aliquot (1 mL) was taken to characterize the PDPES block before adding of St and allowing it to polymerize for another 6 h. After reaction, the mixture was poured into excess methanol to precipitate the polymer, which was then separated through filtration and dried in a vacuum at 50 °C in an oven to constant weight.

### Polymerization of MMA in the presence of PDPES

PDPES (0.14 g, 0.5 mmol) and MMA (2.65 mL, 50 mmol) were added to toluene (2.65 mL). The mixture was stirred at room temperature for 10 min. The bottle was evacuated through three freeze–pump–thaw cycles, purged with purified argon, and then placed in a heated oil bath at 90 °C for 9 h. The resulting polymer was precipitated using ethanol.

### Measurements

^1^H spectra were recorded at 25 °C on a Brucker ARX400 (400 MHz) spectrometer with CDCl_3_ and tetramethylsilane as the solvent and internal reference, respectively.

The molecular weights and molecular weight distributions of the polymers were determined on a Waters gel permeation chromatograph (GPC) equipped with a 515 HPLC pump, 2410 refractive index detector, and three *μ*-Styragel columns (HT3 + HT4 + HT5). All samples were processed in THF at 30 °C at a rate of 1.0 mL/min. Linear PS standards were used for calibration. The data were analyzed using a Waters Millennium 32 system.

The extent of the conversion of the monomer after polymerization was determined through the gravimetric method.

## Results and discussion

### Effect of solvents and temperature on anionic polymerization

**Scheme 1.**
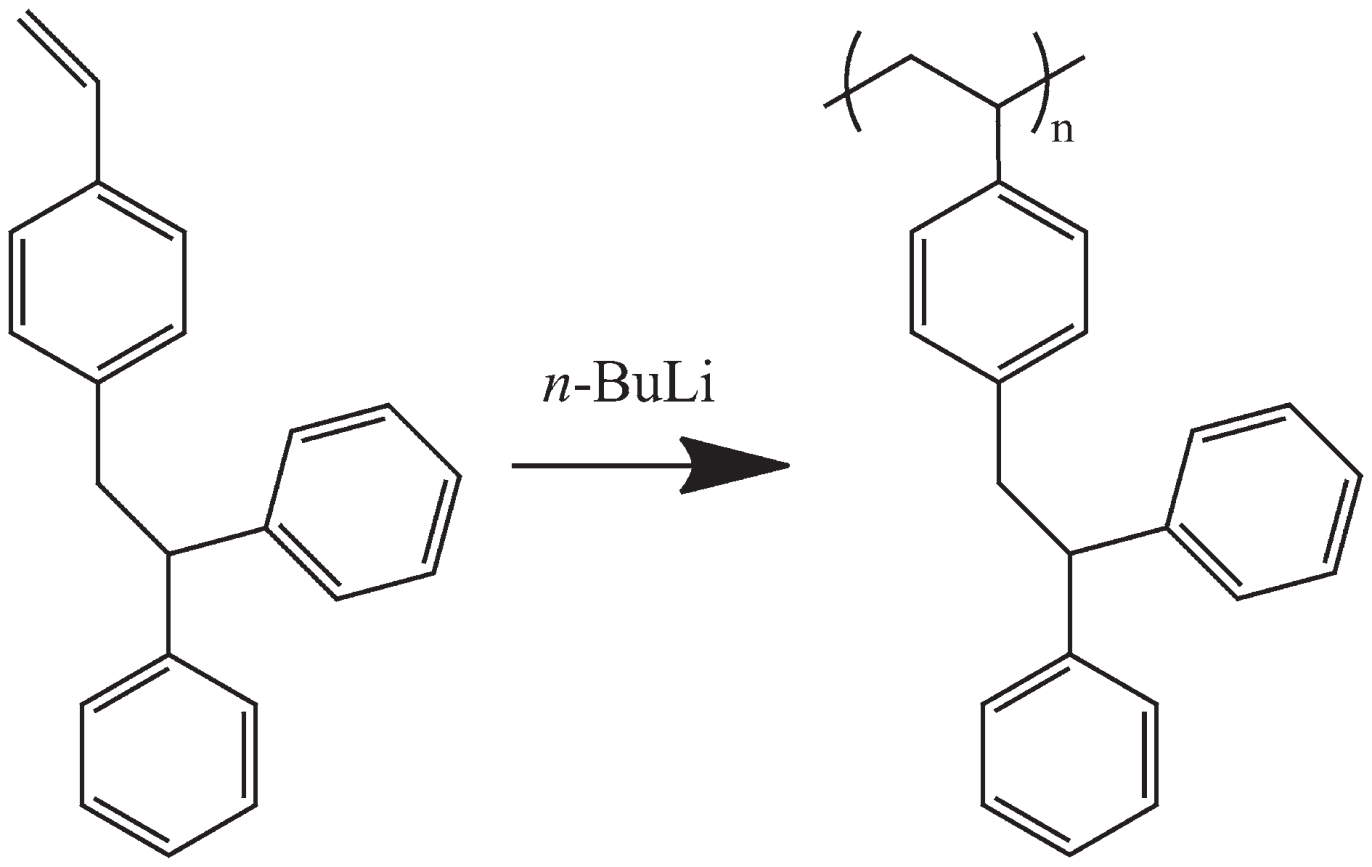
Anionic polymerization of *p*-(2,2′-diphenyle thyl) styrene initiated by *n*-BuLi.

As shown in Scheme 1, DPES has a 2,2′-diphenylethyl group in the para position of the phenyl ring. Because of the large steric hindrance of the bulky TPE structure, the solubility of DPES poorer than St. Meanwhile, the methylene protons adjacent to the aryl are reactive to carbanion in THF. Therefore, it was expected that the anionic polymerization of DPES was difficult to control. To evaluate the controllability for anionic polymerization of DPES, the anionic polymerization of DPES was carried out under different conditions, and the results are displayed in Table 1.

**Figure 1.**
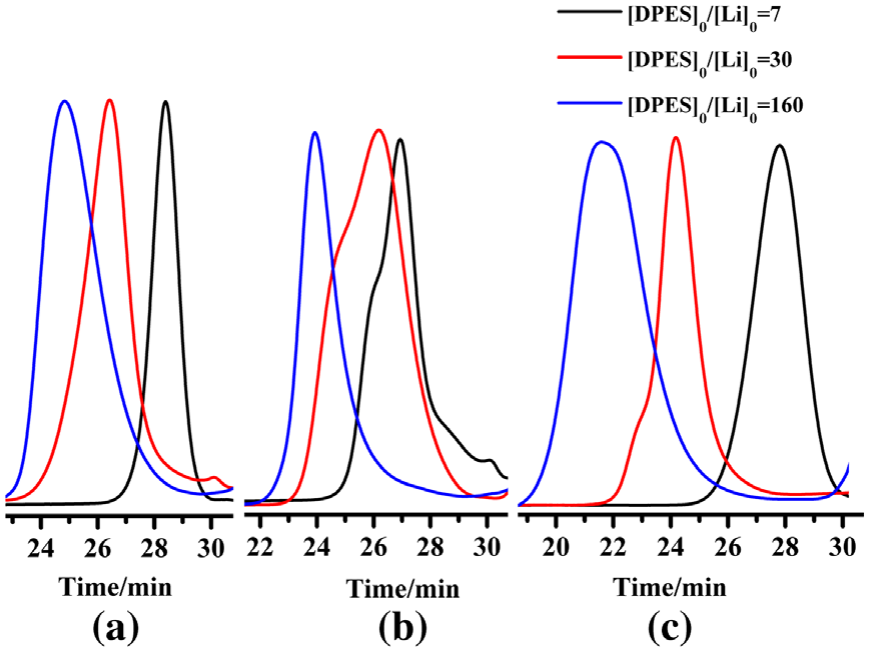
GPC chromatograms of PDPES obtained with different molar ratio of [DPES]_0_/[*n*-BuLi]_0_ at 40 °C in different solvent: (a) cyclohexane, (b) toluene, (c) THF.

When cyclohexane was used as a solvent, the PDPES exhibited a narrow PDI and relatively high yield (Figure 1(a) and Table 1, No. 1). Meanwhile, the *M*_n,GPC_ of PDPES was close to the *M*_n,th_ theoretically calculated based upon the feed ratio of DPES to *n*-BuLi, indicating that no side reactions occurred during the course of the polymerization in cyclohexane. However, PDPESLi precipitated in cyclohexane as a red compound when molar ratio of [DPES]_0_/[*n*-BuLi]_0_ higher than 30, resulting in a lower yield and broader PDI (Table 1, No. 23). To improve the solubility of PDPES in solvent, toluene and THF was used as the solvent of polymerization. When toluene used as solvent, the reaction system solution was opaque during the polymerization process. The Figure 1(b) showed the GPC chromatography analyses of PDPES obtained in toluene. When the molar ratio of [DPES]_0_/[*n*-BuLi]_0_ was 160, the GPC profile showed a single broad peak. When the molar ratio of [DPES]_0_/[*n*-BuLi]_0_ was lower than 30, the GPC profiles showed shoulder peaks, indicating that side reactions seem to be accelerated in toluene. When THF was used as a solvent, the significantly increased yield of PDPES was observed (Table 1, No. 7–9). However, a broad PDI was observed simultaneously (Figure 1(c) and Table 1, No. 7–9). The reason was that THF was a strong complexing agent for *n*-BuLi, which caused unfavorable side reactions, such as transfer reactions, through polarizing C-Li bonds in organolithium compounds. As low-temperature is benefit to eliminate the side reaction, the PDI value of PDPES decreased from 1.26 to 1.09 with the polymerization temperature decreasing to −78 °C (Table 1, No.12).

The above analysis revealed that, the solubility of the propagating species and side reactions are very crucial factors for the well-controlled anionic polymerization of DPES. To improve the solubility of PDPESLi and eliminate side reactions, the mixed solvent of cyclohexane and THF was used for DPES polymerization at 40 °C in a dry argon atmosphere for 6 h. This reaction condition was mild and can be used for the industrial production of polystyrene. The polymerization results obtained are summarized in Table 2.

**Table 2.** Influence of solvent composition on anionic polymerization of DPES^a^.

No.	*V*_cyclohexane_/*V*_THF_	[DPES]_0_/[*n*-BuLi]_0_	Yield (wt％)	*M*_n,th_^b^×10^−3^	*M*_n,GPC_^c^×10^−3^	PDI ^d^
1	1	7	82.5	1.7	1.8	1.20
2	5	7	87.1	2.0	2.4	1.15
3	20	7	100	2.0	2.1	1.08
4	20	14	100	4.0	3.9	1.09
5	20	20	100	5.7	5.7	1.08
6	20	30	100	8.6	8.8	1.09
7	20	160	100	45.5	46.8	1.18

^a^ Polymerization was performed in a dry argon atmosphere at 40 °C for 6 h

^b^ *M*_n.th_: FW(C_22_H_20_) × [DPES]_0_/[*n*-BuLi]_0_ × yield(%) × 10^−2^ + FW(C_4_H_9_)

^c^ *M*_n,GPC_ and

^d^ PDI were estimated using a GPC by employing PS as the standard.

**Figure 2.**
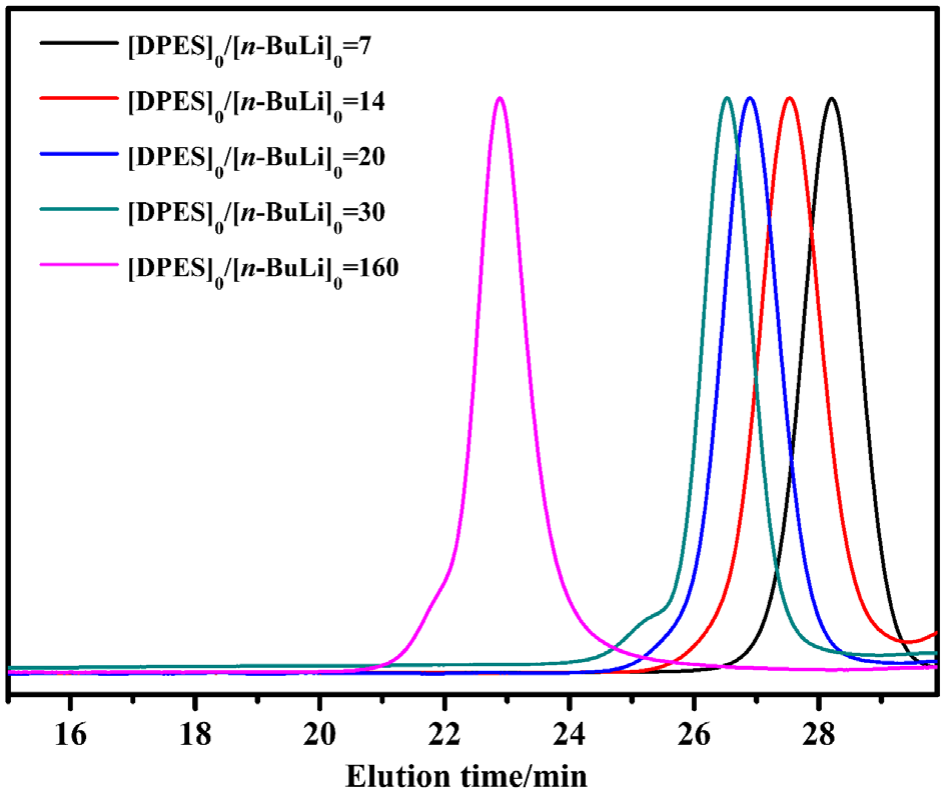
GPC chromatograms of PDPES obtained in mixed solvent of cyclohexane and THF (*V*_cyclohexane_/*V*_THF_ = 20).

When the anionic polymerization of DPES with *n*-BuLi was performed in a mixed solvent (Table 2), each PDPES provided a considerably higher yield of PDPES and narrower PDI value than that in the pure solvent. In particular, the anionic polymerization of DPES in a mixed solvent of cyclohexane and THF (*V*_cyclohexane_/*V*_THF_ = 20) produced PDPES with a 100 wt% yield and the *M*_n_ estimated from the GPC of the resulting PDPES had good agreement with the [DPES]_0_/[*n*-BuLi]_0_ molar ratio (Table 2, Nos. 3–7). As shown in Figure 2, the *M*_n_ of PDPES increased with increasing [DPES]_0_/[*n*-BuLi]_0_ molar ratio, as expected for well-controlled anionic polymerization. Meanwhile, all the GPC chromatograms exhibited narrow PDI value, indicating that unfavorable side reactions were fairly reduced in the mixed solvent (*V*_cyclohexane_/*V*_THF_ = 20). Therefore, for the well-controlled anionic polymerization of DPES at mild temperature, a cyclohexane and THF mixed solvents thought to be required.

**Figure 3.**
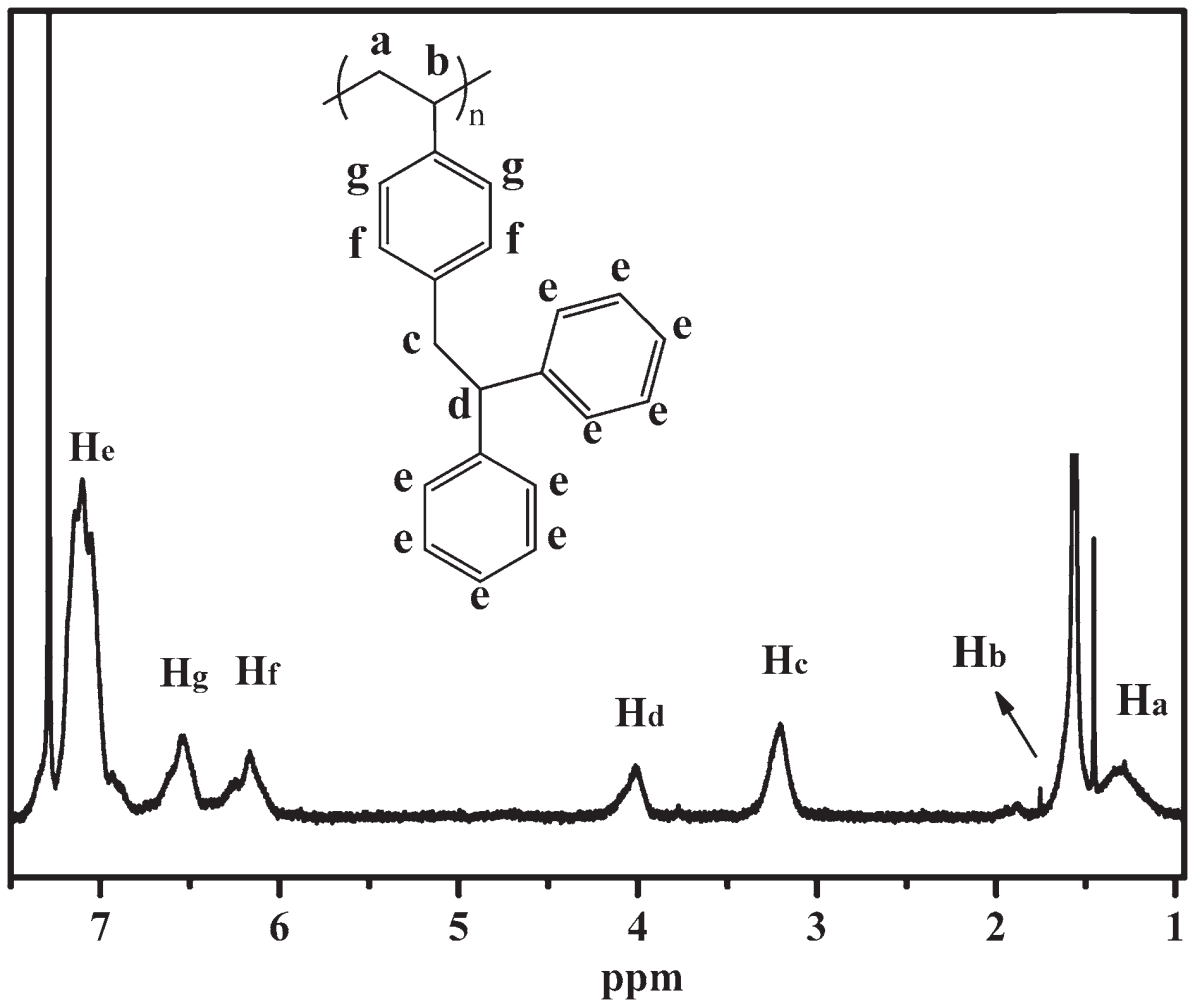
^1^H NMR spectrum of PDPES obtained using *n*-BuLi as initiator in mixed solvent of cyclohexane and THF (*V*_cyclohexane_/*V*_THF_ = 20). (*M*_n_ = 8800, PDI = 1.09), measured in a 3.0 wt% solution of CDCl_3_ at 25 °C.

Figure 3 displays a typical ^1^H NMR spectrum of PDPES synthesized through anionic polymerization with *n*-BuLi in cyclohexane THF mixed solvent (Table 2, No.6). The peaks from 6.0 to 7.3 ppm (He, Hf, Hg) are assigned to aromatic protons in the PDPES. The peaks at approximately 4.0 (Hd) and 3.3 (Hc) are assigned to methine and methylene protons in the PDPES. The peaks at approximately 1.6 ppm (Hb) are assigned to methine protons in the main chain. The peak at approximately 1.3 ppm (Ha) is assigned to methylene protons in the main chain. The ratio of Ha/Hb/Hc/Hd/He/Hf/Hg was 2/1/2/1/10/2/2. The proton ratio strongly supports the polymer chain structure for PDPES that is shown in Figure 3.

### Living nature of the anionic polymerization of DPES with *n*-BuLi in the cyclohexane/THF mixed solvent

From the results summarized in Table 2(Nos.1–7), *n*-BuLi in the cyclohexane/THF solvent appears to cause the well-controlled anionic polymerization of DPES. To further reveal the controlled/living nature, we carried out kinetics experiments. The anionic polymerization of DPES with *n*-BuLi was performed in a cyclohexane/THF mixed solvent (*V*_cyclohexane_/*V*_THF_ = 20) at 40 °C ([DPES]_0_/[*n*-BuLi]_0_ = 56).

**Figure 4.**
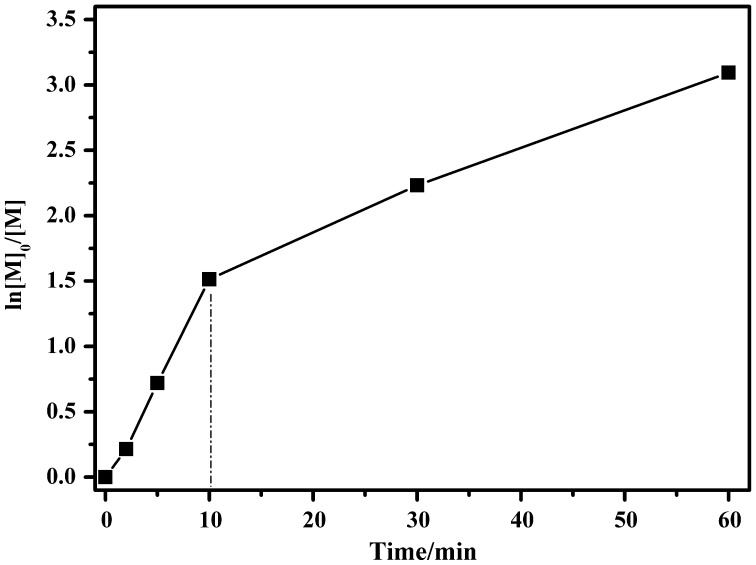
Time–ln([*M*]_0_/[*M*]) plot of the polymerization of DPES using *n*-BuLi as initiator in the cyclohexane/THF mixed solvent (*V*_cyclohexane_/*V*_THF_ = 20) at 40 °C.

**Figure 5.**
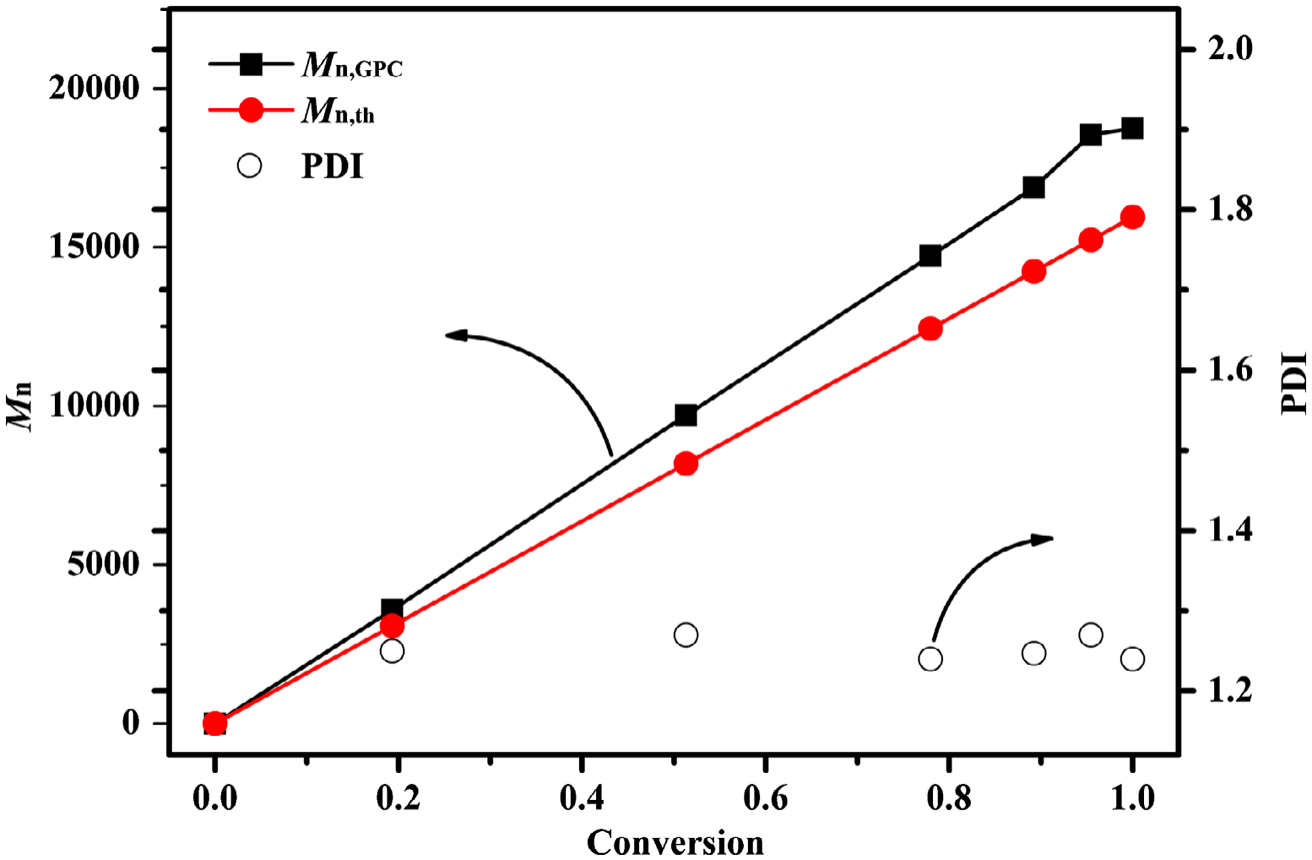
*M*_n_ and PDI of PDPES during the polymerization cyclohexane/THF mixed solvent (*V*_cyclohexane_/*V*_THF_ = 20) at 40 °C.

Figure 4 showed the time–ln([*M*]_0_/[*M*]) plot of the anionic polymerization of DPES. The conversion of DPES was 78% at 10 min. Meanwhile, the plot of time–ln([*M*]_0_/[*M*]) followed a linear relationship within 10 min, indicating constant anionic concentration during this period of time. However, the propagation rate decreased at higher conversion. This decrease in propagation rate can be ascribed to solubility of PDPESLi with high molecular weight in the solvent at high conversion. The *M*_n_ of PDPES during the course of the polymerization increased linearly with the conversion, and all PDI of PDPES were relatively narrow, as expected for a living system (Figure 5). However, the *M*_n,GPC_ of the resulting PDPES was slightly higher than *M*_n.th_, providing further evidence of side reactions at high conversion.

**Figure 6.**
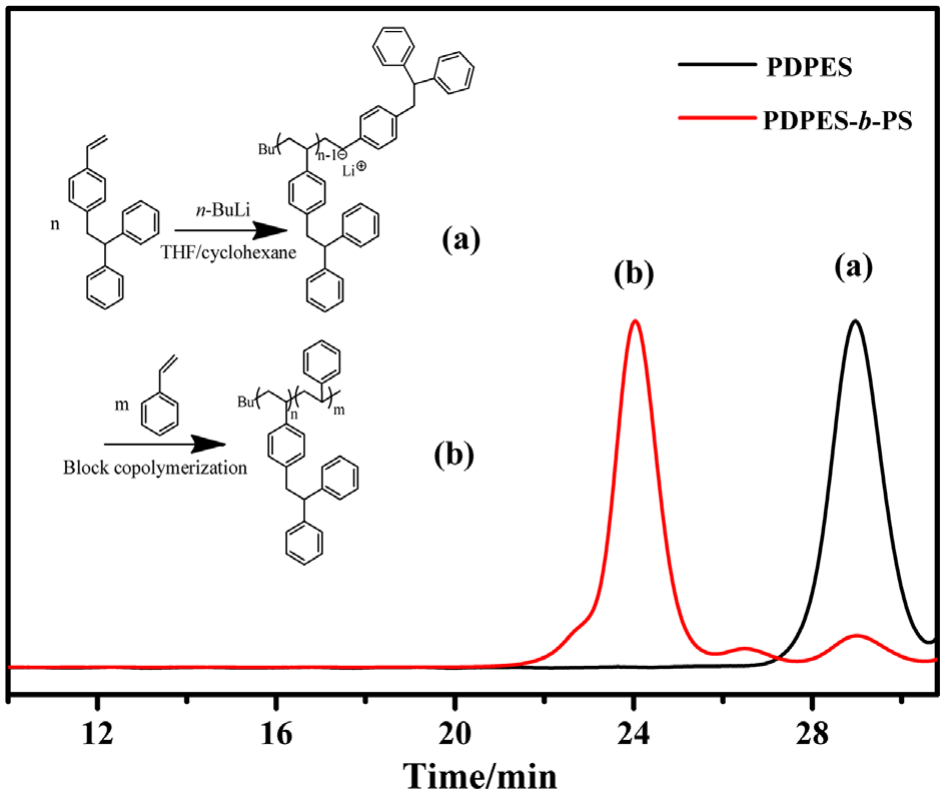
Block copolymerization of DPES and St with *n*-BuLi in the cyclohexane/THF solvent at 40 °C ([DPES]_0_/[St]_0_/[*n*-BuLi]_0_ = 7/300/1). GPC chromatograms of the pre polymer of PDPES (a: *M*_n_ = 1900 g/mol, PDI = 1.14) and the block copolymer of DPES and St (b: PDPES-*b*-PS *M*_n_ = 34,700 g/mol, PDI = 1.17). *M*_n_ and PDI were obtained by GPC calibrated using PS standards.

**Figure 7.**
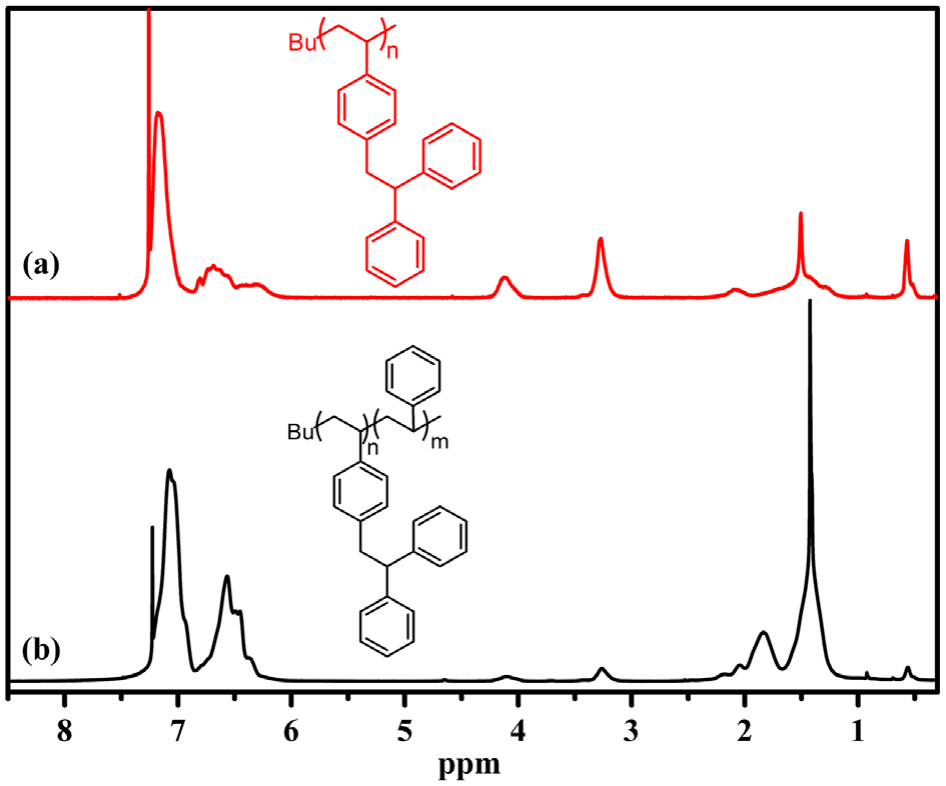
^1^H NMR spectrum of PDPES (a: *M*_n_ = 1900 g/mol, PDI = 1.14) and PDPES-PS block copolymer (b: *M*_n_ = 34 700 g/mol, PDI = 1.17) obtained using the *n*-BuLi as initiator in mixed solvent of cyclohexane and THF (*V*_cyclohexane_/*V*_THF_ = 20), measured in a 3.0 wt% solution of CDCl_3_ at 25 °C.

**Figure 8.**
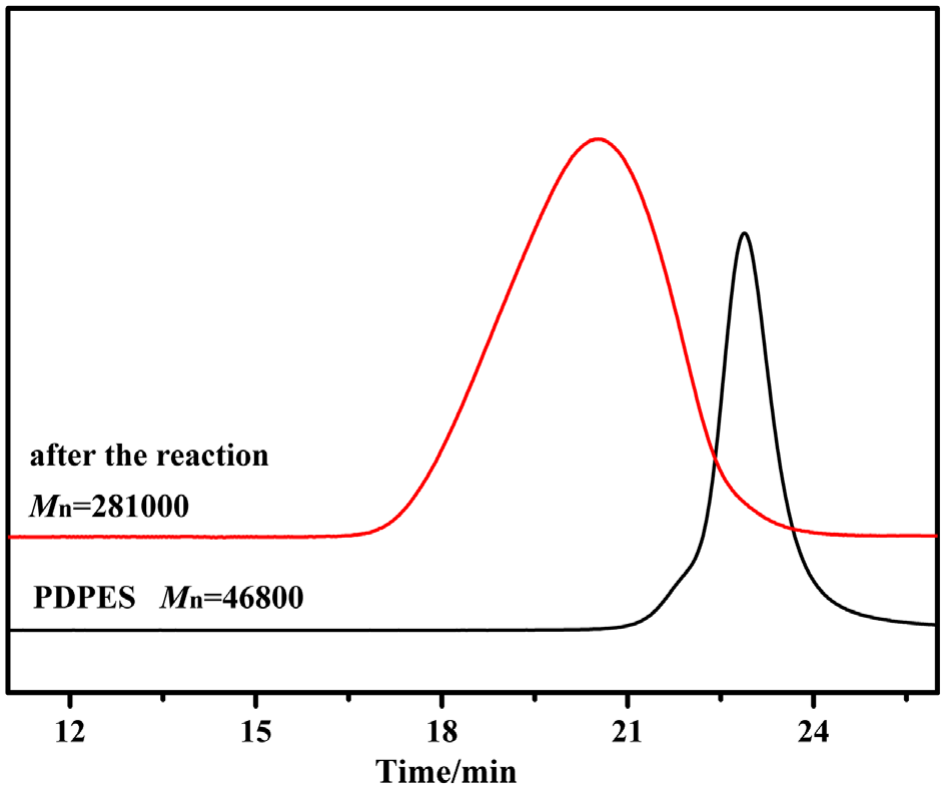
Polymerization of PDPES as macroinitiator with MMA was executed in a dry argon atmosphere at 90 °C for 24 h; GPC chromatograms of PDPES (*M*_n_ = 46,800 g/mol, PDI = 1.18) and PDPES-*g*-PMMA (*M*_n_ = 281,000 g/mol, PDI = 1.86). *M*_n_ and PDI were obtained by GPC calibrated using PS standards.

**Figure 9.**
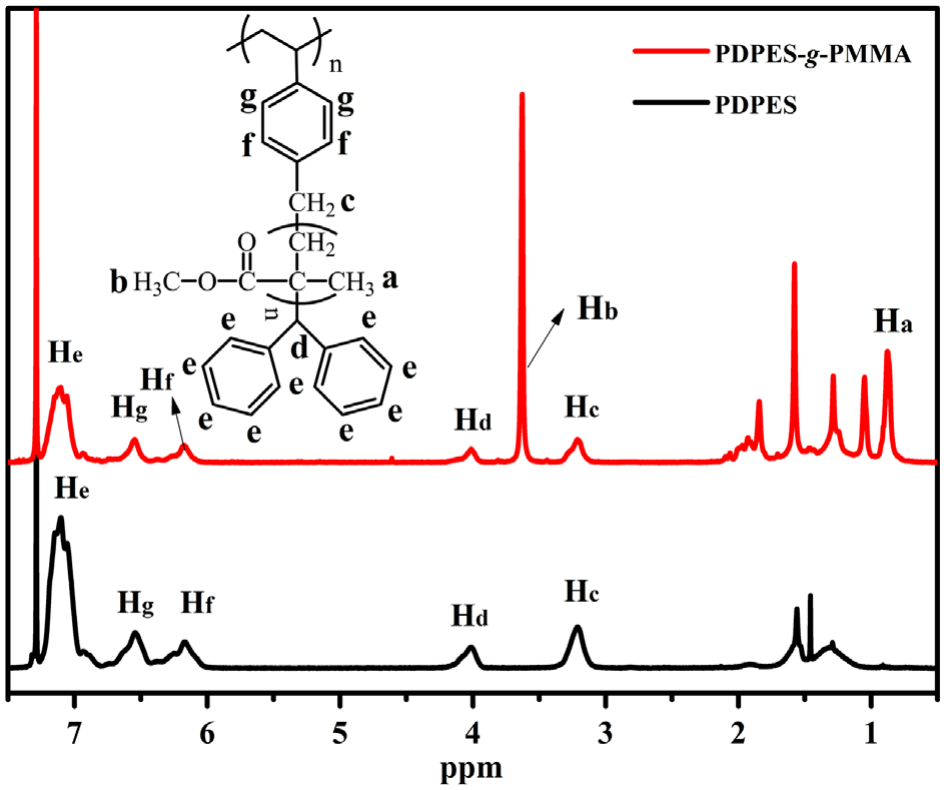
^1^H NMR spectrum of PDPES (*M*_n_ = 46,800, PDI = 1.18) and PDPES- *g*-PMMA (*M*_n_ = 281,000, PDI = 1.86) measured in a 3.0 wt% solution of CDCl_3_ at 25 °C.

**Scheme 2.**
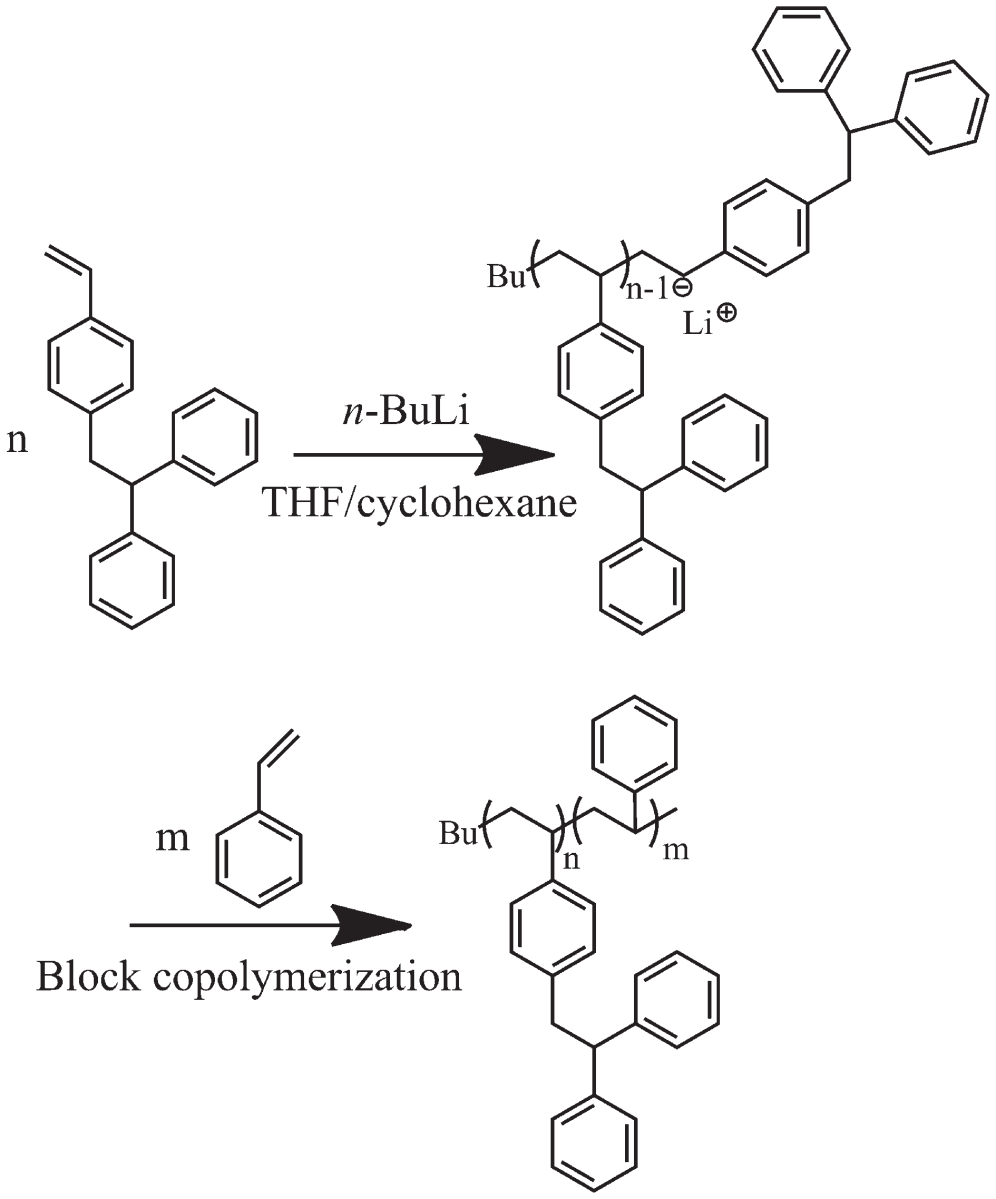
Block copolymerization of DPES and St with *n*-BuLi in the cyclohexane/THF solvent (*V*_cyclohexane_/*V*_THF_ = 20).

The living nature of the polymerization was further examined using a sequential block copolymerization of DPES and St, as shown in Scheme 2. The first step was using *n*-BuLi to initiate the reaction of DPES ([DPES]_0_/[*n*-BuLi]_0_ = 7) in cyclohexane/THF solvent under the protection of an argon atmosphere at 40 °C for 4 h .Subsequently, St was added to the reaction mixture ([St]_0_/[*n*-BuLi]_0_ = 300). The polymerization was continued for 4 h, and 100% conversion was calculated through gravimetric and molecular weight analysis. The copolymer obtained showed a narrow PDI and an obviously increased *M*_n_ from 1900 to 34 700 g/mol (Figure 6). However, the block copolymer contained a small amount of homopolymer. This unpurified block copolymer was probably formed by self-temination of the PDPESLi chains or terminated by St. Meanwhile, the chemical structure of PDPES-*b*-PS was also confirmed by ^1^H NMR spectrum (Figure 7). These results confirmed that PDPES-*b*-PS copolymer was synthesized, indicating that. Therefore, according to the results in Figures 4–7, the polymerization of DPES with *n*-BuLi in a mixed solvent is thought to be a living anionic polymerization process.

### Polymerization of MMA in the presence of PDPES

**Scheme 3.**
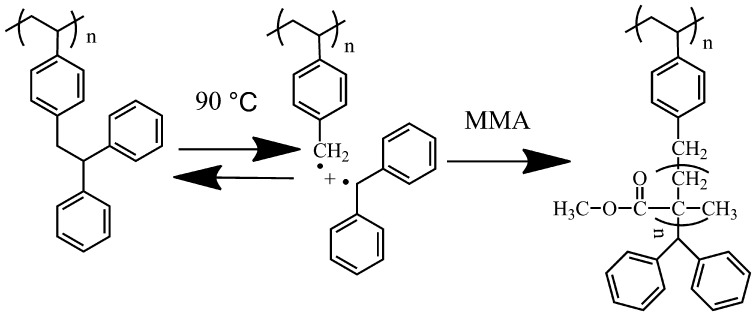
Radical polymerization of MMA with PDPES as a macroinitiator in toluene at 90 °C.

PDPES has an attractive structure because it contains an initiating moiety from a TPE group. Thermal decomposition of PDPES results in an initiating benzyl radical and an inactive diphenylmethyl radical (Scheme 3). This benzyl radical can add to the vinyl group of another monomer (such as MMA) to produce a well-defined graft copolymer.

To demonstrate this deduction, free radical polymerization of MMA with PDPES macroinitiator was performed in toluene at 90 °C for 24 h under the protection of a dry argon atmosphere. Figure 8 shows the typical GPC profiles of the copolymer. The GPC profile of the copolymer that was obtained after the second stage of radical polymerization clearly shifted toward the molecular weight region more than that of the prepolymer of PDPES did, with a relatively broad molecular weight distribution. The broad molecular weight distribution was caused by the nature of radical polymerization and the distribution of the MMA branch on PDPES.

Figure 9 displays the ^1^H NMR spectrum of PDPES and PDPES-*g*-PMMA. The peaks from 6.0 to 7.3 ppm (He, Hg, and Hf) are assigned to aromatic protons in the 2,2′-diphenylethyl and phenyl groups. The peaks at approximately 4.0 ppm (Hd) and 3.3 ppm (Hc) are assigned to methine and methylene protons in the TPE group. The peaks at approximately 3.6 ppm (Hb) and 0.8–1.0 ppm (Ha) are characteristic peaks of PMMA. According to the NMR spectra and GPC profile, we confirmed that the MMA was successfully initiated by the PDPES.

## Conclusion

The anionic polymerization of DPES with *n*-BuLi was examined in detail. The 2,2′-diphenylethyl group strongly affected the DPES solubility and its anionic polymerization behavior. Therefore, the selection of solvent is a crucial factor. The polymerization of DPES in cyclohexane at 40°showed low yield at [DPES]_0_/[*n*-BuLi]_0_ > 30. High conversion was obtained at the same condition when THF was employed, however, the molecular weight distribution widened. The combination of cyclohexane and THF was effective to overcome the problem. When *V*_cyclohexane_/*V*_THF_ was 20, the anionic polymerization of DPES showed living polymerization characteristics. And well-defined block copolymer with St was successfully synthesized. Thermal treatment of PDPES as a macroinitiator in the presence of MMA effectively yielded a graft copolymer composed of a PS backbone and PMMA branches. The macroinitiator could be used to synthesize a variety of sophisticated copolymer and functionalize polymer materials, which will be investigated in our future studies.

## Disclosure statement

No potential conflict of interest was reported by the authors.

## Funding

This work was supported by the National Natural Science Foundation of China [grant number 51473010]; National Basic Research Program of China [grant number 2015CB654701].
